# Report of Three Cases in Which Unusual Symptoms, Etc.

**Published:** 1853-04

**Authors:** 


ART. IV.—*Report of three cases in which unusual symptoms were present.*

[The following communication was received without date or signature.—*Ed.*]

Mr. Editor:—As much of the interest of your *Journal* depends upon reports of rare cases, and since many rare cases are unlikely to occur in the practice of one Physician, I have thought it proper to send you a history of the following three, which, if you think of sufficient importance, you may may insert in your *Journal.*

Case 1.—In March, 1848, I was called to see Mrs. C., aged 39; light hair; grey eyes; slender constitution; had been married several years; had never been pregnant. Her health for the last few years had been feeble, the catamenia irregular, and sometimes profuse. I found her with a frequent and feeble pulse; tongue slightly coated, no present pain. She informed me that her abdomen for some time previous had been somewhat larger than usual; but that a few hours before my arrival, a large gush of limpid fluid had escaped from the vagina, on which the abdominal walls became flaccid. This fluid continued to drain away for some three weeks, at the end of which time it ceased, leaving the woman, however, much debilitated. The amount passed was sufficient to saturate from 6 to 12 napkins a day.

As I had never seen a similar case before, I was at a loss as to what course to pursue. I gave however, alumn internally, alternating its use with acetate of lead, followed by carb. iron, which was well borne. Solutions of these articles were injected per vaginum. After she got about, her health was much as before, for two years, when she died of typhus fever.

CASE 2.—In May, 1851, I was called to Mrs. M., at two o'clock in the morning, of whose case the following history was given: At ten o'clock on the day before, she was taken in labor with her sixth child. It was soon, discovered by the women present, that the right arm presented. In a short time, the pains died away, to the sorrow of the sapient midwife. A dispute now arose as to the best course to pursue. Some were for letting things alone till the services of a physician could be procured. But the old midwife assured them that a few more pains would do the work; and consequently her feet were put in hot water; hot teas given, and in fact everything was done to start the pains, which soon came on, and continued for ten hours, not light, but powerfully and severe. When it was found that she was not going to get through, I was sent for. A short time before my arrival, she felt something give way in the abdomen. I found her bedewed with a cold sweat, the child's arm had been forced low down, and the shoulder firmly impacted in the bones. I called for a light, but it was brought in time only to see our patient die. Her husband, and all but the midwife, thought her sacrificed by an untimely resort to stimulants. And who believes but that she might have been saved, had not nature been lashed into action when she was waiting for help? No examination was made, but there was probably laceration of the uterus.

CASE OF LINGERING PARTURITION.

CASE 3.—On the morning of the 15th inst., I was called to Mrs. B., aged 40; light hair; blue eyes; small size; large pelvis; and had borne nine children. On the morning of the 13th, she told me there had been a gush of water from the vagina; and as all her former labors had been quite rapid, she soon expected to be through. The women were called in, and pains came on once in about ten minutes, until I was called. I could feel the child's head low down; the os easily admitting two fingers, and feeling confident that her pains must increase in force before delivery, I strove to quiet her fears of an unfortunate result. I left after remaining twelve hours, and in two days called again. Pains continued as before, coming on regularly every ten or fifteen minutes. Little, if any descent had taken place in the child. I began to think her mistaken about the discharge of waters four days before. She insisted that she could not have been mistaken, for the abdomen had suddenly shrunk with the discharge of the fluid.

I did not see my patient again for ten days, when on being called to her, I found her just delivered of a small, living, plump, female child. Her pains from the first continuing regular, were only light, and most of the time she was about the house, or sitting on the sofa. No fever occurred; in fact there was nothing calling for interference, save costiveness. So far as I know, such cases are quite rare, as no Physician in my acquaintance has seen one similar.

